# Dynamic Inter-Modality Source Coupling Reveals Sex Differences in Children based on Brain Structural-Functional Network Connectivity: A Multimodal MRI Study of the ABCD Dataset

**DOI:** 10.1101/2025.07.23.666366

**Published:** 2025-07-29

**Authors:** A. Kotoski, S-L. Wiafe, J. M. Stephen, Y-P. Wang, T. W. Wilson, V. D. Calhoun

**Affiliations:** 1 Tri-Institutional Center for Translational Research in Neuroimaging and Data Science, Atlanta, GA, USA; 2 Neuroscience Institute, Georgia State University, Atlanta, GA, USA; 3 Department of Computer Science, Georgia State University, Atlanta, GA, USA; 4 Mind Research Network, Albuquerque, NM, USA; 5 Department of Biomedical Engineering, Tulane University, New Orleans, LA, USA; 6 Institute for Human Neuroscience, Boys Town National Research Hospital, Omaha, NE, USA; 7 Department of Pharmacology and Neuroscience, Creighton University, Omaha, NE, USA

**Keywords:** Structure-function coupling, Sex differences, Dynamic functional connectivity

## Abstract

**Background:**

Sex differences in brain development have been widely reported in both structural and functional domains, particularly during late childhood and adolescence. Prior studies have shown that males and females differ in gray matter volume, network connectivity profiles, and their associations with behavior and cognition. However, how these sex differences manifest in the coupling between brain structure and function remains less understood. In this study, we introduce dynamic inter-modality source coupling (dIMSC), an extension of our earlier inter-modality source coupling method (IMSC). While IMSC evaluates the coupling between source-based morphometry (SBM) from structural MRI (sMRI) and static functional network connectivity from resting-state fMRI (rs-fMRI), dIMSC incorporates the temporal dimension by linking SBM with dynamic functional network connectivity (dFNC).

**Objectives:**

This study investigated the transient coupling between dynamic FNC (dFNC) and sMRI gray matter volume over time, and compared sex differences in dFNC-sMRI coupling across brain regions in children.

**Methods:**

We used data from the Adolescent Brain Cognitive Development (ABCD) study, focusing on children aged 9–11 years. Structural MRI data were analyzed using SBM, applying independent component analysis (ICA) to extract gray matter sources. Resting-state fMRI data were processed to compute dFNC using a sliding window approach. For each subject, dIMSC was computed as the cross-correlation between the dFNC matrix and the SBM vector, resulting in a time-resolved vector that reflects the strength of structure-function coupling across components. Coupling values were categorized into positive, neutral, or negative based on a specific threshold. Sex differences in dFNC-sMRI coupling were evaluated using two-sample t-tests with correction for multiple comparisons.

**Results:**

Our analysis revealed significant sex-specific patterns, with males exhibiting stronger positive coupling in the postcentral gyrus and precuneus, whereas females showed stronger coupling in the inferior parietal lobule and middle frontal gyrus. Additional sex differences emerged in the neutral and negative coupling domain, with males demonstrating stronger coupling in the superior temporal gyrus, calcarine gyrus, and superior parietal lobule, whereas females exhibited stronger coupling in the caudate nucleus, cerebellum, and inferior parietal lobule.

**Conclusion:**

Together these findings suggest distinct coupling in brain structure-function coupling between sexes, potentially reflecting sex-specific organization of functional networks and their structural substrates. The dIMSC method advances our earlier work by enabling time-resolved analysis of brain structure-function coupling, providing a powerful framework for investigating neurodevelopmental processes.

## I. INTRODUCTION

Sex differences in brain development, historically underexamined in neuroscience, are now receiving increased attention due to their potential role in shaping cognitive, behavioral, and psychiatric trajectories [1–3]. From early childhood through adolescence, males and females exhibit differences in brain maturation rates, cortical thickness, connectivity, and functional organization [4, 5]. Prior studies have reported sex-specific patterns in brain volume, white matter development, and resting-state network activity [4, 6, 7], with some suggesting that these differences may influence emotional regulation, or susceptibility to disorders such as attention-deficit/hyperactivity disorder (ADHD), anxiety, and autism [8–10]. However, while many of these findings highlight structural or functional differences between sexes, most of the literature has examined these domains in isolation, with limited insight into how brain structure and function interact across sexes during development. Therefore, the mechanisms by which structural-functional coupling differ between males and females remain largely unexplored.

Structural MRI (sMRI) and resting-state functional MRI (rs-fMRI) are widely used to study the brain during development and to evaluate sex differences [4, 5]. sMRI provides information into gray and white matter anatomy, including cortical volume, thickness, and surface area [11]. Functional MRI (fMRI) is used to indirectly study the brain’s activity by measuring the changes in blood-oxygen levels [12]. rs-fMRI applies this technique at rest capturing the connectivity of brain regions when no task or stimulus is being applied [13]. Prior research has demonstrated sex-related differences in both modalities: males often show larger total brain volumes, and females exhibit a larger volume in areas related to language [14]. Resting-state studies revealed sex differences in network connectivity, particularly within the default mode, dorsal attention network, and the salience network [15]. Although these findings suggested that sex modulates both brain structure and function, they often treated each modality separately. What remains unclear is how structure-function relationships may differ between sexes.

To address this gap, it is crucial to investigate how structural and functional brain features interact dynamically. In a prior study, we introduced inter-modality source coupling (IMSC) [16], a method that quantified the association between structural and functional brain components derived from independent component analysis (ICA) [17]. While IMSC has provided insights into static structure-function coupling, it does not account for temporal fluctuations that may reflect dynamic brain activity. To address this limitation, we propose a novel extension called dynamic inter-modality source coupling (dIMSC) which allows for time-resolved assessment of structure-function coupling. To capture the temporal evolution of brain function, we use dynamic functional network connectivity (dFNC) derived from sliding-window based Pearson correlation, which estimates time-varying connectivity between functional brain networks [18, 19]. dFNC provides a more nuanced representation of brain activity by capturing transient brain states that are obscured when averaging over longer periods, as is done in static FNC. Some of these dynamic states may be more closely aligned with underlying brain structure than others, making dFNC a more sensitive measure for detecting structure-function coupling. For brain structure, we utilize the source-based morphometry (SBM) components—spatially independent gray matter sources derived from ICA—to capture patterns of covarying structural variation across individuals [20]. dIMSC is computed by correlating SBM components with each temporal snapshot of dFNC pattern, enabling us to track how structural and functional interactions evolve over time. By applying dIMSC to a sample of children, we aim to explore if there is a structure-function coupling difference between males and females.

Using dIMSC, we found that functional brain states differ in their relationship to brain structure, with distinct patterns of positive, neutral, and negative coupling states. On average, males showed slightly higher proportions of positive coupling, while females showed more negative coupling. Regionally, females exhibited greater coupling in the inferior parietal lobule and middle frontal gyrus during the positive state, while males showed stronger coupling in sensorimotor and parietal regions during the neutral and negative states. These findings highlight sex-specific dynamics in how structural and functional brain networks interact during late childhood, revealing developmental mechanisms that may underlie cognitive and behavioral differences.

## II. METHODS

In this study, we use a novel method to examine time-varying interactions between brain structure and function in children aged 9–11 years-old. We analyzed baseline data from the ABCD study, which includes over 11,000 children recruited from 21 sites across the United States. The sample is demographically diverse and was designed to approximate the U.S. population in terms of age, sex, ethnicity, and socioeconomic status [21]. This approach captures transient coupling between dynamic functional network connectivity (dFNC) and structural MRI gray matter volume over time. We then use it to explore sex differences in dFNC-sMRI coupling across brain regions during this critical developmental period.

### A. Dynamic Inter-Modality Source Coupling

We used a standardized functional template consisting of multiple replicable resting fMRI brain networks, estimated via ICA [17], as spatial priors for both structural and functional modalities. This approach ensured consistency and comparability across subjects while also adapting to each subject individually. Specifically, we used fully automated spatially constrained ICA (scICA), applied separately to rs-fMRI and sMRI data, to extract subject-specific components while preserving spatial independence [16]. This enabled us to evaluate cross-modal coupling within a unified framework, where rs-fMRI captures individual dynamic patterns and structural brain morphometry reflects inter-subject covariation. We used the 53-component NeuroMark_fMRI_1.0 template [22] as the spatial prior, ensuring that the resulting components were both data-adaptive and comparable across modalities. All analyses were conducted using the GIFT toolbox [23].

For rs-fMRI data, we computed a dynamic functional network connectivity matrix using sliding window Pearson correlation (SWPC) [24], which estimates time-varying functional interactions. The analysis employed a rectangular window of 44s with a step size of 1 TR [18, 19], allowing us to capture fluctuations in connectivity over time. This approach provides a dynamic view of functional organization beyond static connectivity measures by characterizing how interactions between brain networks evolve temporally.

For sMRI analysis, we applied constrained SBM to estimate subject-specific loading parameters for 53 brain regions [16]. SBM is a data-driven multivariate approach that identifies spatially independent structural patterns and quantifies their expression in individual subjects [25]. Using gray matter volume as input, we extracted independent components that capture covarying patterns across subjects. Each subject’s structural profile was represented by a 53-component loading vector, where each component reflects the degree to which the corresponding gray matter pattern is expressed in that individual.

To link the two modalities within our proposed dIMSC approach, we estimate the network expression coupling between the dFNC pattern and the SBM pattens via cross-correlation, computed at each time step 𝑡 across 304 sliding windows between the dFNC matrix and the corresponding SBM vector for each subject:

$$dFNC\left(t\right)\;\epsilon \;{\mathbb{R}}^{53X53}$$

$$SBM\;\epsilon \;{\mathbb{R}}^{53X1}$$

$$dIMSC\left(t\right)=corr\left(dFNC\left(t\right),\;SBM\right)$$

Where:

- $dIMSC\left(t\right)\;\epsilon \;{\mathbb{R}}^{53X1}$ is the dynamic inter-modality source coupling at time t for each subject.
- $dFNC\left(t\right)\;\epsilon \;{\mathbb{R}}^{53X53}$ is the dynamic functional connectivity matrix at time t for each subject.
- $SBM\;\epsilon \;{\mathbb{R}}^{53X1}$ is the structural component vector for each subject.

**Figure 1.**
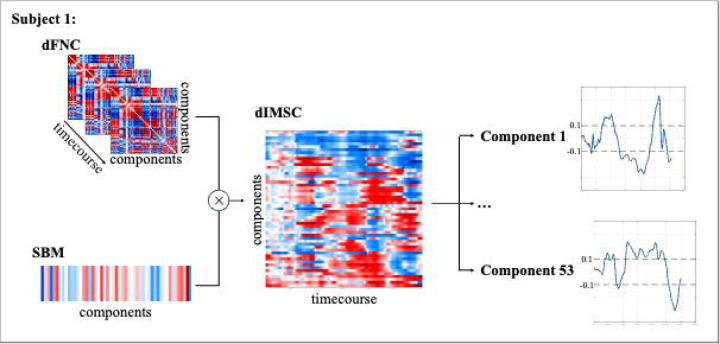
Overview of the dIMSC pipeline for a representative subject. The NeuroMark functional template was used as a prior in constrained ICA applied to resting-state fMRI and structural MRI data to extract 53 subject-specific functional and structural components. For rs-fMRI, a sliding window-based Pearson correlation was used to compute dFNC matrices. For sMRI, SBM was computed to generate a structural vector with 53 components. At each timepoint, dIMSC was computed as the cross-correlation between the dFNC matrix and the SBM vector, resulting in a time-resolved vector that reflects the strength of the structure-function coupling across components. The resulting matrix was decomposed into 53 dIMSC components and each timepoint based on a correlation threshold for each of the 53 brain networks based on the distribution percentiles of the values.

This correlation results in a timecourse that indicates how the functional characteristics of the network (represented as a column in the dFNC matrix) align with the expression of covarying gray matter components (represented as a row in the SBM mixing matrix) in each subject. This analysis resulted in a matrix that showed the degree of dynamic structure-function coupling. Figure 1 shows the pipeline for the dIMSC method. Subsequently, we categorized each timepoint based on a correlation threshold for each of the 53 brain networks based on the distribution percentiles of the dIMSC values. We selected a threshold of ρ=±0.1, such that approximately 50% of the timepoints fall into a neutral group, while the positive and negative groups each contain approximately 25%. This distribution aligns with the expected behavior of coupling, where fluctuations around a stable mean are more common, and larger deviations occur less frequently [26]. This ensures that the positive and negative groups reflect meaningful changes in coupling while maintaining balanced subgroup sizes. On average, across all participants, 27.12% of timepoints exhibited positive coupling, 46.62% were classified as neutral, and 26.26% displayed negative coupling. For each category of the degree of time-resolved (or dynamic) structure-function coupling values, we computed the occupancy rate, representing the proportion of time a network spends in a particular coupling state.

The neutral category correlation values near zero represents timepoints where the dynamic inter-modality structure-function coupling is weak. Importantly, this category should not be interpreted as a distinct or meaningful coupling state, but rather as reflecting a stable period without significant alignment between structural and functional patterns. It serves as a reference against which deviations towards stronger positive or negative coupling can be identified or characterized. By distinguishing these neutral timepoints, we can better capture meaningful fluctuations in coupling dynamics while accounting for period of relative stability.

$$C_{i}\left(t\right)=\left\{\begin{array}{cc} Positive & if\;dIMSC\left(t\right)>0.1\\ Neutral & if-0.1\leq dIMSC\left(t\right)\leq 0.1\\ Negative & dIMSC\left(t\right)<-0.1 \end{array}\right.\;\;OR_{i,c}=\frac{\sum_{t=1}^{T}1\left(C_{i}\left(t\right)=c\right)}{T}$$

where:

- $OR_{i,c}$ is the occupancy rate for category c in brain network i.
- T is the total number of timepoints.
- $1\left(C_{i}\left(t\right)=c\right)$ is an indicator function that equals 1 if $C_{i}\left(t\right)$ is in category c and 0 otherwise.

To examine sex differences in dIMSC measures, we conducted a multiple linear regression including a regressor for sex (male vs female) and, site and intracranial volume, computed using FreeSurfer v6.0 [27], as a nuisance regressor. A significance threshold of p<.05 was used, with corrections for multiple comparisons applied using the false discovery rate (FDR).

### B. Data

As noted above, the Adolescent Brain Cognitive Development (ABCD) study [28] the longitudinal development of children during a 10-year study using a multisite design. To reduce computational demand, we selected a random subset of 500 unrelated individuals, matched for age, to capture meaningful effects while maintaining feasibility for analysis. The female groups consisted of 245 participants, aged between 107 months and 132 months (mean = 119 months; SD = 7 months). The male group consisted of 255 participants, aged between 107 months and 132 months (mean = 132 months; SD = 7 months). Demographic and scanner-related characteristics for each group are summarized in Table 1.

### C. MRI Parameters

Data were collected on three types of 3T scanners (Siemens Prisma, General Electric 750 and Philips) all with a standard adult-size 32-channel head coil. The MRI sequences acquired and used for this work were a T1 structural scan (TR = 2500/2500/6.31 ms respectively; TE = 2.88/2/2.9 ms respectively). The resting-state EPI sequence used the following parameters: TR = 800 ms, TE = 30 ms, 60 slices, flip angle = 52°, matrix size = 90 × 90, FOV = 216 × 216 mm, resolution = 2.4 × 2.4 × 2.4 mm. The full details of the imaging acquisition protocol are described in [28].

## III. RESULTS

The application of dIMSC revealed distinct temporal patterns of structure-function coupling across the 53 brain networks. By estimating the coupling between the network expression of the dFNC matrix and the SBM vector at each timepoint, we obtained a time-resolved measure of coupling strength. These values were grouped into three categories: positive, neutral, and negative coupling. Specifically, positive coupling indicates that increases in structural properties are associated with increases in functional properties, suggesting a coordinated relationship; neutral coupling indicates that structural and functional properties are relatively independent of each other; and negative coupling implies an inverse relationship, potentially indicating compensatory or opposing mechanisms between the areas.

**Figure 2.**
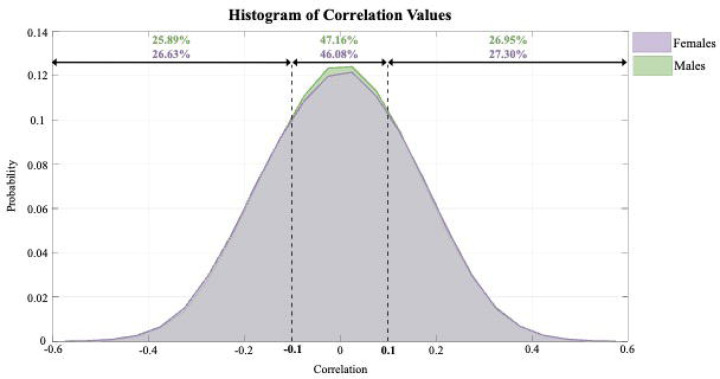
This histogram displays the distribution of coupling for males (on the back, shown in green), and females (on the front, shown in purple). The x-axis represents the correlation value between structure and function, while the y-axis indicates the probability of distributions. Vertical dashed lines are placed at −0.1 and 0.1 along the x-axis to define the three groups: negative, neutral and positive. These thresholds highlight a clear group-level difference, where around 50% of the values are concentrated in the neutral group. For the females, this distribution suggests a greater proportion of negative and positive values when compared to males. In contrast, the male’s correlation values are more centered around the neutral group, indicating that males tend to exhibit more neutral values on this coupling.

The mean coupling strength was 0.18 ± 0.04 for positive states, 0.0001 ± 0.02 for neutral states, and −0.17 ± 0.04 for negative states. This distribution indicates a predominant trend of balanced structure-function alignment across the sample, with stronger positive and negative coupling suggesting a bidirectional relationship between structural and functional features in specific networks. When analyzed separately by sex, males exhibited positive coupling in 26.96% of timepoints, neutral coupling in 47.16%, and negative coupling in 25.89%. The corresponding mean coupling strengths were 0.18 ± 0.04 for positive states, 0.0002 ± 0.02 for neutral states, and −0.17 ± 0.04 for negative states. In females, positive coupling was observed in 27.30% of timepoints, neutral coupling in 46.08%, and negative coupling in 26.63%, with mean coupling strengths of 0.17 ± 0.04, 5.43 × 10^−6^ ± 0.02, and −0.17 ± 0.04, respectively. These results indicate that males showed a slightly higher proportion of timepoints with neutral coupling, while females exhibited a marginally greater proportion of negative and positive coupling. Figure 2 illustrates the distribution of the coupling for males and females. On average, males had a total intracranial volume of 1583.55 ± 119.96 cm^3^, while females had an average intracranial volume of 1438.48 ± 191.21 cm^3^. To account for potential head size effects, all statistical comparisons were adjusted for intracranial volume.

**Figure 3.**
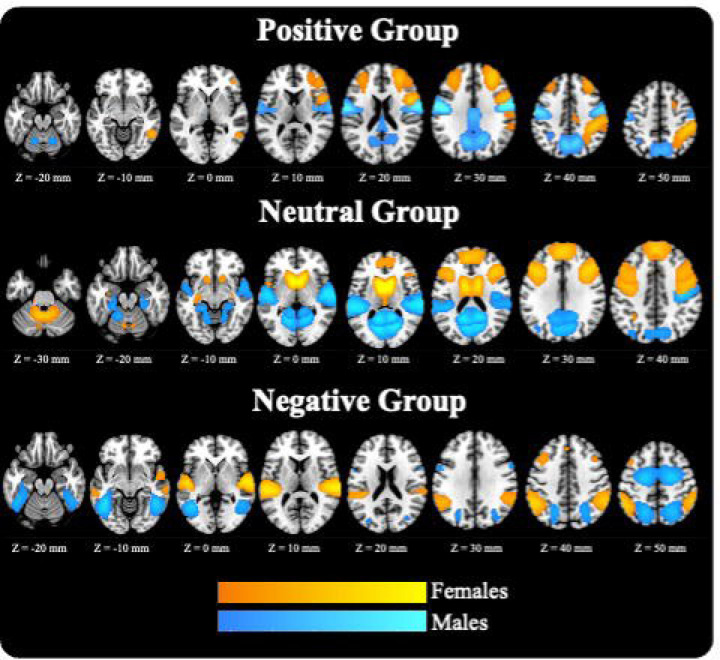
Brain maps showing significant sex differences in structure-function coupling across categories. Regions with significant group differences are displayed separately for positive group (top), neutral group (middle), and negative (bottom). Males are shown in blue and females in orange, allowing for visual comparison between groups. Anatomical localization is shown in MNI space.

**Figure 4.**
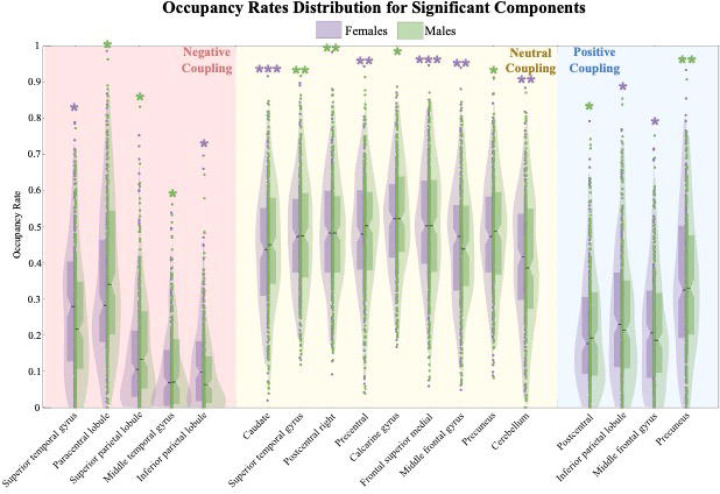
This figure presents group-level violin plots displaying state-specific occupancy rates for brain regions that exhibited significant sex differences, with values shown separately for males (green) and females (purple). The y-axis represents the occupancy rate, defined as the proportion of timepoints during which each brain region was classified within a specific coupling state. The x-axis lists the individual brain regions, grouped into three coupling states: negative coupling (left, shaded red), neutral coupling (center, shaded yellow), and positive coupling (right, shaded blue). Occupancy rates reflect how often a region engaged in a given coupling profile over time. In the positive coupling category, females showed stronger coupling than males in the inferior parietal lobule and precuneus, suggesting enhanced structural support for functional activity. In the neutral coupling category, males exhibited stronger coupling in the cerebellum and superior temporal gyrus, whereas females showed stronger coupling in the caudate, precentral gyrus, and middle frontal gyrus, pointing to potential sex-specific stability in these regions. In the negative coupling category, males showed stronger coupling in the paracentral lobule, superior parietal lobule, and middle temporal gyrus, while females showed stronger coupling in the superior temporal gyrus and inferior parietal lobule. Violin plots display the full distribution of occupancy values, with overlaid individual data points and wider sections indicating higher density. Asterisks indicate statistically significant group differences (*.05 > p > .01, **.01 > p > .001, ***p < .001), corrected for multiple comparisons using FDR. Notably, sex differences appear relatively evenly distributed across all three coupling states. This suggests that sex differences in brain activity patterns occur throughout the full range of brain network dynamics. This pattern might reflect that biological sex influences the coordinated brain activity in general, regardless of the specific coupling strength.

Sex differences in dIMSC were observed across several brain networks. Figure 3 provides spatial brain maps highlighting the identified regions, while Figure 4 presents the occupancy rate values for significant brain regions in both males and females. Within the positive coupling category, females exhibited stronger coupling in the inferior parietal lobule and precuneus compared to males, suggesting enhanced structural support for functional activity in these regions. In the neutral coupling category, males demonstrated stronger coupling in the cerebellum and superior temporal gyrus, while females exhibited stronger coupling in the caudate, precentral, and middle frontal gyrus, suggesting that different brain regions may underlie sex-specific structure-function coupling during this state. For the negative coupling category, males showed stronger coupling in the paracentral lobule, superior parietal lobule, and middle temporal gyrus, while females exhibited stronger coupling in the superior temporal gyrus ad inferior parietal lobule.

## IV. DISCUSSION

In this study, we introduced a new method, dynamic inter-modality source coupling, to investigate the time-varying relationship between brain structure and function and applied to the ABCD dataset. Extending our previous IMSC method [16], dIMSC captures dynamic patterns of coupling between gray matter structural components and dynamic functional connectivity across 53 brain networks. Unlike traditional static methods, which offer only a snapshot of structure-function relationships, dIMSC leverages correlation and source separation techniques to model how structural components dynamically align or decouple from evolving patterns of functional connectivity. Applied across 53 large-scale brain networks derived from multimodal imaging data, this approach allows us to identify distinct coupling states—positive, neutral, and negative—at regional levels.

We grouped coupling values at each timepoint into positive, neutral, or negative categories, based on correlation thresholds derived from the distribution of dIMSC values. This grouping approach acknowledges that structure-function relationships in the brain are not fixed but fluctuate over time. These fluctuations may indicate state-dependent shifts in functional engagement or neural efficiency. For example, periods of stronger (positive) coupling reflects a strong alignment between brain structure and function, suggesting that functional connectivity is effectively supported by the underlying structural components. Neutral coupling indicates little to no correlation between structure and function. This may represent moments when brain function operates more independently of structural components that are detectable noninvasively. Negative coupling suggests an inverse relationship between structure and function, which may reflect a reciprocal relationship, in which functional dynamics temporarily diverge from structural constraints [26]. Overall, we found that approximately 27% of timepoints exhibited positive coupling, 47% were neutral, and 26% negative, with similar proportions across sexes.

In the positive coupling state, females showed greater structure-function coupling in the inferior parietal lobule (IPL) and middle frontal gyrus, and males showed greater coupling in the postcentral gyrus and precuneus. The IPL is a key region involved in multimodal integration, attention, and social cognition [29]. Stronger coupling in this region among females may reflect earlier maturation or greater integration of associative regions, consistent with previous findings indicating accelerated structural and functional development in girls during late childhood [30]. Similarly, the middle frontal gyrus, part of the dorsolateral prefrontal cortex and a region implicated in executive functions, showed greater coupling in females. This may suggest a developmental trend toward enhanced structure-function alignment in networks supporting executive processing, aligning with prior reports that females tend to outperform males on tasks involving cognitive control and working memory in this age range [1, 31]. In contrast, stronger coupling in males was observed in the postcentral gyrus and precuneus—regions associated with sensorimotor integration and self-referential processing [31]. While speculative, this pattern may reflect sex differences in how internal and external sensory information is integrated during this developmental stage. Although our current analysis does not directly link coupling strength to behavioral performance, these findings may point to underlying neural substrates contributing to sex-differentiated cognitive and sensory development. Future studies incorporating ABCD cognitive and behavioral measures will be important and helpful for clarifying the functional relevance of these observed differences.

The neutral state, which contains the largest proportion of timepoints, is characterized by overall weak coupling between structural and functional components. Despite the low absolute coupling strength, we observed that males exhibited relatively stronger coupling than females in sensorimotor and visual areas, including the superior temporal gyrus, postcentral gyrus, calcarine cortex, and precuneus. While neutral coupling reflects a weak dynamic relationship between structure and function, the presence of consistently stronger neutral coupling in these regions for males may suggest a form of structural anchoring. In other words, even when these regions are not actively engaged in synchronized functional dynamics, the underlying structural architecture may continue to exert a stabilizing influence on their functional potential. This interpretation aligns with prior work indicating that sensorimotor and visual networks tend to mature earlier in development [32]. Females, showed stronger coupling in higher-order associative and motor planning areas, including the caudate, precentral gyrus, superior medial frontal gyrus, and middle frontal gyrus, and cerebellum. The caudate, known for its role in goal directed behavior and reward processing, has previously shown sex differences in structure and function, with females often exhibiting greater activation and gray matter volume [33, 34]. While caution is warranted in interpreting values from the neutral range, these differences may reflect more consistent structural support for cognitive control and motor planning networks in females during this developmental window.

Negative coupling reflects functional configurations that are opposite or reciprocal to the underlying structural architecture. In this state, males showed stronger negative coupling in the paracentral lobule, superior parietal lobule, and middle temporal gyrus—regions involved in motor and visuospatial processing. This may indicate a functional pattern that diverges from structural connectivity, possibly representing flexible or dynamic functional states that are not strictly constrained by anatomy. Similarly, females exhibited stronger negative coupling in the superior temporal gyrus and inferior parietal lobule, regions linked to language, social cognition, and multisensory integration. These patterns might reflect an intrinsic reciprocal relationship where functional interactions transiently oppose structural pathways, supporting complex and flexible brain function during development. Future work, including comparisons with sMRI and fMRI brain age measures, could help elucidate these opposing structure-function dynamics.

The observed sex differences in dynamic structure-function coupling underscore the importance of considering sex as a biological variable in developmental neuroimaging studies. These findings suggest that males and females may differ not only in static brain measures but also in how their brains dynamically link anatomy and function over time. These coupling patterns may reflect underlying neurodevelopmental mechanisms, possibly related to synaptic pruning, myelination, and network integration, processes known to vary by sex [6, 35, 36].

By demonstrating that dynamic structure-function coupling can be quantified and meaningfully interpreted in children, our work contributes to studies on multimodal integration and the use of both spatial and temporal information in neuroscience. The dIMSC approach provides a scalable, interpretable approach to investigate sex differences in brain development, providing insights into how structural and functional networks interact dynamically during late childhood. While this study emphasized sex differences, the approach can be extended to explore individual trajectories associated with neurodevelopmental conditions, such as ADHD and autism, which often exhibit sex-biased prevalence and distinct symptom profiles in males and females [9, 10]. Future studies are needed to directly test the hypothesis that negative structure–function coupling reflects a reciprocal or opposing relationship between functional dynamics and underlying structural architecture, whereas positive coupling reflects alignment between the two modalities. Integrating behavioral and cognitive measures in future research will be essential to clarify the functional significance of these opposing structure-function patterns. Longitudinal studies could help determine whether these patterns are transient or stable features of development, and individual variability in coupling strength might be influenced by environmental, genetic, or hormonal factors.

## V. CONCLUSIONS

This study introduced dIMSC, a novel method to capture time-varying relationships between brain structure and function across 53 brain networks in children. By leveraging dynamic functional connectivity and structural gray matter volume components, dIMSC provides a temporally resolved measure of structure-function coupling that goes beyond traditional static approaches. Our findings revealed distinct coupling states—positive, neutral, and negative—reflecting fluctuating patterns of alignment, independence, and inverse relationships between structural and functional brain properties. These dynamic coupling patterns exhibited subtle but meaningful sex differences across multiple brain regions, including associative, sensorimotor, and subcortical areas. Females showed stronger positive coupling in regions implicated in executive control and integration, while males exhibited greater coupling in sensorimotor and visuospatial regions, suggesting sex-specific trajectories of brain maturation and network organization.

These results highlight that brain structure-function relationships in childhood are dynamic and influenced by biological sex, emphasizing the need to incorporate temporal and multimodal perspectives in developmental brain imaging. The dIMSC approach offers a powerful framework to investigate how evolving structural and functional networks interact to support neurodevelopment, with potential applications to studying typical and atypical brain maturation. Future longitudinal studies integrating behavioral, genetic, and hormonal data will be essential to help elucidate the developmental mechanisms underlying these dynamic coupling patterns and their links to cognitive and clinical outcomes. Overall, our work advances understanding of the complex, time-dependent interplay between brain anatomy and function during a critical period of childhood development and lays the groundwork for investigating individual differences and neurodevelopmental disorders.

## Figures and Tables

**Table 2.** Overview of demographic and scanner-related characteristics by sex. IQ scores reflect the uncorrected NIH Toolbox total composite scores. Parental education represents the highest level of education reported by either parent, with “college degree or higher” defined as completion of a bachelor’s degree, master’s degree, professional degree, or doctoral degree. Parental employment indicated whether at least one parent was employed full-time. Household income was grouped into three categories, low (<$35,000), middle ($35,000-%99,999), and high (≥$100,000). Race/ethnicity was reported by parents and categorized as White, Black or African American or Other (including Hispanic, Asian, and multiracial individuals).

Variables	Female (n = 245)	Male (n = 255)
**IQ (mean** ± **SD)**	85.86 ± 13.07	85.50 ± 10.35
**MRI Scanner Site**	60.3% Siemens 14.8% Philips 24.8% GE	62.0% Siemens 12.4% Philips 25.6% GE
**Parental Education**	18.6% high school degree or less 50.0% college degree or higher	18.5% high school degree or less 59.3% college degree or higher
**Parental Employment**	73.5% full-time employed	74.8% full-time employed
**Household Income**	19.4% low 36.4% middle 38.8% high	22.5% low 26.7% middle 45.3% high
**Race/Ethnicity**	57.0% White 9.1% Black or African American 33.9% other	48.4% White 12.4% Black or African American 39.2% other
